# Differentiating coronavirus disease 2019 (COVID-19) from influenza and dengue

**DOI:** 10.1038/s41598-021-99027-z

**Published:** 2021-10-05

**Authors:** Tun-Linn Thein, Li Wei Ang, Barnaby Edward Young, Mark I-Cheng Chen, Yee-Sin Leo, David Chien Boon Lye

**Affiliations:** 1 National Centre for Infectious Diseases, 16 Jalan Tan Tock Seng, Singapore, 308442 Singapore; 2 Department of Infectious Diseases, Tan Tock Seng Hospital, 11 Jalan Tan Tock Seng, Singapore, 308433 Singapore; 3 Lee Kong Chian School of Medicine, Nanyang Technological University, 11 Mandalay Road, Singapore, 308232 Singapore; 4 Saw Swee Hock School of Public Health, National University of Singapore and National University Health System, 12 Science Drive 2, #10-01, Singapore, 117549 Singapore; 5 Yong Loo Lin School of Medicine, National University of Singapore and National University Health System, 10 Medical Drive, Singapore, 117597 Singapore

**Keywords:** Dengue virus, Influenza virus, SARS-CoV-2

## Abstract

The novel coronavirus disease 2019 (COVID-19) presents with non-specific clinical features. This may result in misdiagnosis or delayed diagnosis, and lead to further transmission in the community. We aimed to derive early predictors to differentiate COVID-19 from influenza and dengue. The study comprised 126 patients with COVID-19, 171 with influenza and 180 with dengue, who presented within 5 days of symptom onset. All cases were confirmed by reverse transcriptase polymerase chain reaction tests. We used logistic regression models to identify demographics, clinical characteristics and laboratory markers in classifying COVID-19 versus influenza, and COVID-19 versus dengue. The performance of each model was evaluated using receiver operating characteristic (ROC) curves. Shortness of breath was the strongest predictor in the models for differentiating between COVID-19 and influenza, followed by diarrhoea. Higher lymphocyte count was predictive of COVID-19 versus influenza and versus dengue. In the model for differentiating between COVID-19 and dengue, patients with cough and higher platelet count were at increased odds of COVID-19, while headache, joint pain, skin rash and vomiting/nausea were indicative of dengue. The cross-validated area under the ROC curve for all four models was above 0.85. Clinical features and simple laboratory markers for differentiating COVID-19 from influenza and dengue are identified in this study which can be used by primary care physicians in resource limited settings to determine if further investigations or referrals would be required.

## Introduction

A cluster of cases of pneumonia with unknown cause was detected in Wuhan in December 2019^1^. Caused by a novel human coronavirus now named severe acute respiratory syndrome coronavirus 2 (SARS-CoV-2), it has since spread rapidly on a global scale. On 11 March 2020, the World Health Organization (WHO) declared the novel coronavirus disease (COVID-19) as a pandemic, and expressed deep concern about the alarming levels of spread and severity^2^. Confirmation of acute SARS-CoV-2 infection requires detection of the virus in respiratory samples by reverse transcriptase polymerase chain reaction (RT-PCR) test. Clinical presentation of COVID-19 patients ranges from asymptomatic to mild non-specific acute symptoms such as fever, dry cough and fatigue^3^. Close to 20% of COVID-19 cases may be severe^4^.

Influenza is prevalent globally and remains an important cause of morbidity and mortality from respiratory viral infections^5^ while dengue is prevalent in tropical countries with geographic expansion^6^. Dengue is also known as not only the leading cause of fever in returning travelers in non-endemic countries, but also the main source for triggering autochthonous transmissions^7,8^. Since clinical presentations of these common viral infections are non-specific, it is difficult for primary care physicians to differentiate COVID-19 from influenza and dengue. This may result in misdiagnosis or delayed diagnosis, and lead to further transmission in the community. As the COVID-19 pandemic progresses, doctors in both dengue endemic^9–11^ and non-endemic countries where influenza may be the most relevant differential diagnosis for COVID-19^5^,^12^, should maintain a high level of suspicion and be provided with effective tools to differentiate these three infections.

Singapore is a tropical city state with several endemic viral infections. Influenza circulates year round with bimodal peaks typically observed in April-July and November-January^13^. Dengue epidemics of increasing magnitude have occurred in a five- to six-year cycle affecting more adults than children^14^. As of 15 June 2020, a total of 40,818 cases of the COVID-19 including 26 deaths have been reported in Singapore^15^. It is of concern that two local cases of COVID-19 with false positive dengue serology were reported in Singapore without travel or contact history^16^.

In this study, we compared clinical presentations and laboratory parameters of patients with COVID-19, influenza and dengue. We constructed predictive models using logistic regression with the aim to assist doctors in differentiating COVID-19 from influenza and dengue based on clinical features and simple laboratory investigations to determine if further investigations or referrals would be required.

## Methods

### Cohort description

Our study population comprised laboratory confirmed COVID-19 from an ongoing prospective cohort, and influenza and dengue patients from previous studies. Briefly, prospective recruitment of COVID-19 patients took place from January to April 2020 at the National Centre for Infectious Diseases (NCID), which is a purpose-built facility designed to strengthen Singapore’s capabilities in infectious disease management^3^. All COVID-19 suspect cases, i.e., clinical signs and symptoms suggestive of pneumonia or severe respiratory infection with breathlessness or acute respiratory illness of any severity, were sent to NCID and other hospitals via dedicated ambulances for testing and isolation during the study period.

Influenza patients were selected from a retrospective study of those who presented at Tan Tock Seng Hospital (TTSH), the primary centre for screening, treatment and isolation of influenza A(H1N1)pdm09 cases from April to June 2009 in Singapore^17^. Dengue patients were selected from a prospective cohort study where adult patients presented with acute undifferentiated febrile illness to TTSH from January 2010 to September 2012^18^.

### Data collection and confirmatory testing

At first presentation to the hospital, demographic data, symptoms and signs were collected. Full blood count, renal and liver function tests were performed. SARS-CoV-2, influenza and dengue viruses were detected in respiratory samples or venous blood by RT-PCR tests^3,17,18^. Data of 215 patients with COVID-19 (all with wild-type strain), 200 with H1N1(pdm09) or seasonal influenza, and 300 with dengue (predominantly dengue virus serotype 2) were extracted. As our objective was to derive early predictors to differentiate COVID-19 from influenza and dengue, we analysed only symptomatic patients who presented to hospital within 5 days after symptom onset.

### Ethics consideration

The study procedures were carried out in accordance with relevant guidelines and regulations. Ministry of Health (MOH) and the National Healthcare Group Domain Specific Review Board (NHG DSRB), Singapore approved the studies (A Multi-centred Prospective Study to Detect Novel Pathogens and Characterize Emerging Infections (E/12/917), Prospective adult dengue study (E/09/432) and Characterization of influenza in local setting including novel influenza A 2009 cases (E/09/344)). Data for COVID-19 patients were collected with waiver of informed consent granted by MOH under the Infectious Diseases Act as part of COVID-19 outbreak investigation. Research samples and data for the prospective COVID-19 and dengue cohort studies were collected with written informed consent approved by NHG DSRB. Data from influenza patients were collected with waiver of informed consent approved by NHG DSRB as stated earlier.

### Statistical analysis

Fisher’s exact test was used to compare categorical variables and Mann–Whitney U test to compare continuous variables between any two groups. All statistical tests were two-sided, and statistical significance was taken as *P* < 0.05.

We used multivariable logistic regression models as predictive tools to differentiate between COVID-19 and influenza, and COVID-19 and dengue. We followed the transparent reporting of a multivariable prediction model for individual prognosis or diagnosis (TRIPOD) 2015 guideline^19^.

Two multivariable models were fitted for differentiating COVID-19 versus influenza (flu models 1 and 2) and COVID-19 versus dengue (dengue models 1 and 2): flu model 1 and dengue model 1 contained demographics and symptoms, whereas flu model 2 and dengue model 2 included laboratory parameters in addition to demographics and symptoms.

For the analysis on COVID-19 versus influenza, the proportion of missing data for laboratory parameters ranged from 3.4% (10 out of total 297 observations) for WBC count, haemoglobin, platelet, neutrophil count and lymphocyte count, to 28.3% (84 observations) for albumin, alanine aminotransferase (ALT) and aspartate aminotransferase (AST). The variables for flu models 1 and 2 were selected through stepwise use of Akaike’s information criterion. Multicollinearity was then checked using variance inflation factor (VIF) for the final predictor variables included in these 2 multivariable models, which ranged from 1.01 to 1.25.

For the analysis on COVID-19 versus dengue, the proportion of missing data for laboratory parameters ranged from 3.3% (10 out of total 306 observations) for WBC count, haemoglobin, haematocrit, platelet, neutrophil count and lymphocyte count, to 9.2% (28 observations) for AST. As the data for differentiating COVID-19 versus dengue exhibited separation, finite estimates of adjusted odds ratios (aOR) and Wald confidence intervals (CI) could not be obtained. Hence, we applied Firth’s modified score procedure to estimate OR and derived CI using the profile-penalized likelihood function^20^. The variables in dengue models 1 and 2 were selected by backward stepwise approach using penalized likelihood ratio tests. The VIF for the final predictor variables ranged from 1.01 to 1.23 for dengue model 1, and it ranged from 1.31 to 3.67 in dengue model 2 when laboratory parameters were included.

We assessed the predictive performance of the multivariable logistic regression models using receiver operating characteristic (ROC) curves and the corresponding area under the ROC (AUC). As the evaluation of the predictive performance of a set of independent variables using the original sample may yield an overly optimistic estimate of how the results would generalize to an independent set of data, we applied a tenfold cross-validation to obtain a more realistic estimate of AUC for each model.

## Results

This study comprised 126 patients with COVID-19, 171 with influenza and 180 with dengue, who presented within 5 days after symptom onset. The demographic characteristics of the patients are shown in Table 1. The median age of COVID-19 patients was older compared with influenza and dengue patients. A lower proportion of COVID-19 patients were male compared with dengue patients. The proportion of COVID-19 patients having comorbidities was higher compared with dengue patients but not with influenza patients.

**Table 1:** Demographics of COVID-19, influenza and dengue patients.

	COVID-19 (N = 126)	Influenza (N = 171)	*P*-value	Dengue (N = 180)	*P*-value
Age, median (IQR)	52 (37–62)	30 (22–40)	< 0.0005	36 (29–43)	< 0.0005
Male, n (%)	74 (58.7)	98 (57.3)	0.813	153 (85.0)	< 0.0005
**Co-morbidity^, n (%)**	26 (20.6)	31 (18.1)	0.655	18 (10.0)	0.012
Diabetes	15 (11.9)	1 (0.6)	< 0.0005	1 (0.6)	< 0.0005
Chronic obstructive pulmonary disease (COPD)/Asthma	4 (3.2)	8 (4.7)	0.569	0 (0.0)	0.027
Myocardial infarction	7 (5.6)	–	–	1 (0.6)	0.009
Malignancies	2 (1.6)	–	–	0 (0.0)	0.167
Chronic liver disease	1 (0.8)	–	–	0 (0.0)	0.410
Chronic renal disease	1 (0.8)	–	–	0 (0.0)	0.410

*IQR* interquartile range, ‘- ‘ indicates data is not available.

All *P* values shown are for comparison with COVID-19, based on Fisher’s exact test for categorical variables and Mann–Whitney U test for continuous variables.

^None of the COVID-19 and dengue patients had congestive heart failure, peripheral vascular disease, stroke or dementia, while these comorbid conditions were not recorded for influenza patients.

The clinical features of patients with COVID-19, influenza and dengue at presentation are shown in Table 2. Shortness of breath and diarrhoea were more common in COVID-19 patients than in influenza patients, while fever, cough, running nose and sore throat were less common. Cough, shortness of breath, running nose and sore throat were more common in COVID-19 patients than in dengue patients. A lower proportion of COVID-19 patients had fever, diarrhoea, muscle aches, fatigue/malaise, abdominal pain, bleeding, conjunctivitis, headache, joint pain, skin rash and vomiting/nausea compared with dengue patients. We also provided an infographic of percentage of COVID-19, influenza and dengue patients with each symptom at presentation. It can be seen that COVID-19 and influenza patients have similar symptoms, while dengue patients present with symptoms that are significantly different from the other two groups (See Supplementary Table S1).

**Table 2:** Clinical features at presentation of COVID-19, influenza and dengue patients.

Clinical feature, n (%)	COVID-19 (N = 126)	Influenza (N = 171)	*P*-value	Dengue (N = 180)	*P*-value
Fever	86 (68.3)	155 (90.6)	< 0.0005	180 (100.0)	< 0.0005
Cough	82 (65.1)	139 (81.3)	0.002	44 (24.4)	< 0.0005
Shortness of breath	17 (13.5)	1 (0.6)	< 0.0005	8 (4.4)	0.005
Running nose	30 (23.8)	97 (56.7)	< 0.0005	14 (7.8)	< 0.0005
Sore throat	48 (38.1)	97 (56.7)	0.002	39 (21.7)	0.002
Diarrhoea	16 (12.7)	2 (1.2)	< 0.0005	68 (37.8)	< 0.0005
Muscle aches	26 (20.6)	31 (18.1)	0.655	137 (76.1)	< 0.0005
Fatigue/malaise	12 (9.5)	8 (4.7)	0.107	161 (89.4)	< 0.0005
Abdominal pain	1 (0.8)	3 (1.8)	0.64	32 (17.8)	< 0.0005
Bleeding	0 (0.0)	–	–	56 (31.1)	< 0.0005
Chest pain	2 (1.6)	–	–	10 (5.6)	0.132
Conjunctivitis	0 (0.0)	–	–	8 (4.4)	0.023
Headache	13 (10.3)	29 (17.0)	0.129	153 (85.0)	< 0.0005
Joint pain	1 (0.8)	–	–	111 (61.7)	< 0.0005
Skin rash	0 (0.0)	–	–	54 (30.0)	< 0.0005
Vomiting/nausea	5 (4.0)	8 (4.7)	1.000	130 (72.2)	< 0.0005

*OR* odds ratio, *CI* confidence interval, ‘-‘ indicates data is not available.

All *P* values shown are for comparison with coronavirus disease 2019 (COVID-19), based on Fisher’s exact test.

The vital signs and laboratory parameters of patients with COVID-19, influenza and dengue are shown in Table 3. COVID-19 patients had lower white blood cell (WBC) count, neutrophil count and creatinine compared with influenza patients whereas their lymphocyte count and alanine aminotransferase (ALT) were higher. WBC, platelet, neutrophil and lymphocyte counts and albumin were higher in COVID-19 patients than dengue patients. The levels of haemoglobin, haematocrit, aspartate aminotransferase (AST) and creatinine were lower in COVID-19 patients than dengue patients.

**Table 3:** Vital signs and laboratory parameters of COVID-19, influenza and dengue patients.

Value, median (IQR)	COVID-19 (N = 126)	Influenza (N = 171)	*P*-value	Dengue (N = 180)	*P*-value
**Vital signs at presentation**					
Temperature, °C	37.7 (37.1–38.1)	38.2 (37.6–38.7)	< 0.0005	37.5 (37.0–38.1)	0.547
Heart rate, beats per minute	89.0 (80.0–99.8)	103 (95–115)	< 0.0005	75 (66–84)	< 0.0005
Respiratory rate, breaths per minute	18.0 (17.0–19.0)	–	–	18 (18–18)	0.715
Systolic blood pressure (mmHg)	133.0 (121.0–146.0)	111 (103–123)	< 0.0005	119 (110–128)	< 0.0005
Diastolic blood pressure (mmHg)	80.0 (73.0–88.0)	68 (60–75)	< 0.0005	72 (65–80)	< 0.0005
Pulse oximeter O2 saturation (%)	98.0 (97.0–99.0)	–	–	99 (98–100)	< 0.0005
**Baseline laboratory investigations**					
WBC count, × 10^9^/L	4.7 (3.9–5.9)	6.8 (5.6–8.0)	< 0.0005	2.5 (2.0–3.2)	< 0.0005
Haemoglobin, g/dL	14.2 (13.0–15.1)	14.0 (13.0–14.9)	0.342	15.1 (14.1–15.9)	< 0.0005
Haematocrit, %	42.1 (38.7–44.4)	–	–	44.0 (41.4–46.6)	< 0.0005
Platelet count, × 10^9^/L	200.5 (168.5–237.5)	210.0 (171.0–260.0)	0.066	97.5 (73.0–119.0)	< 0.0005
Neutrophil count, × 10^9^/L	2.7 (2.0–4.1)	5.0 (3.5–6.3)	< 0.0005	1.5 (1.1–2.0)	< 0.0005
Lymphocyte count, × 10^9^/L	1.2 (0.9–1.5)	0.9 (0.7–1.3)	< 0.0005	0.6 (0.4–0.8)	< 0.0005
Albumin, g/L	41.0 (38.0–43.0)	40.0 (38.0–42.0)	0.463	39.0 (37.0–42.0)	0.044
ALT, U/L	28.0 (19.0–44.8)	20.0 (15.0–32.5)	< 0.0005	31.0 (22.0–52.0)	0.062
AST, U/L	26.5 (19.0–36.3)	24.0 (20.0–30.0)	0.271	47.0 (32.0–77.8)	< 0.0005
Creatinine, µmol/L	70.0 (59.0–89.0)	82.0 (67.3–98.0)	< 0.0005	76.0 (68.0–88.8)	0.011

*IQR* interquartile range, *ALT* alanine aminotransferase, *AST* aspartate aminotransferase, *WBC* white blood cell.

‘-‘ indicates data is not available.

All *P* values shown are for comparison with coronavirus disease 2019 (COVID-19), based on Mann–Whitney U test for continuous variables.

**Figure 1:**
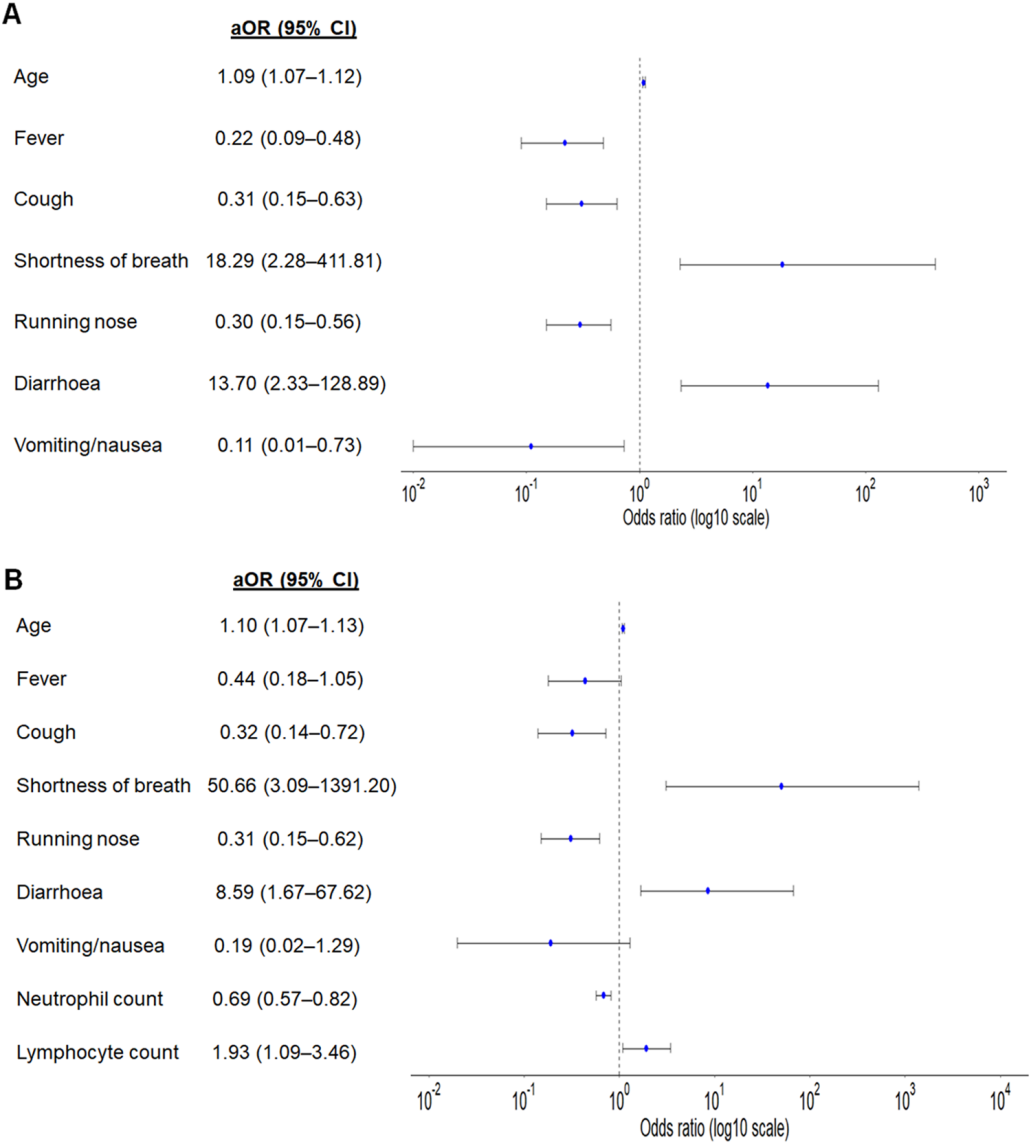
Final covariates in multivariable logistic regression for differentiating COVID-19 versus influenza: (**A**) model 1 on demographics and symptoms, (**B**) model 2 on demographics, symptoms and laboratory Adjusted odds ratios (aOR) and 95% confidence interval (CI)l for having COVID-19 are shown on log10 scale.

The multivariable logistic regressions differentiating COVID-19 from influenza are shown in Fig. 1. In flu model 1 containing demographics and symptoms, older age (aOR 1.09; 95% CI 1.07–1.12), shortness of breath (aOR 18.29; 95% CI 2.28–411.81) and diarrhoea (aOR 13.70; 95% CI 2.33–128.89) increased the odds that the patient had COVID-19, while fever, cough, running nose and vomiting/nauseas were indicative of influenza. In flu model 2 containing demographics, symptoms and laboratory parameters, older age (aOR 1.10; 95% CI 1.07–1.13), shortness of breath (aOR 50.66; 95% CI 3.09–1391.20), diarrhoea ((aOR 8.59; 95% CI 1.67–67.62) and higher lymphocyte count (aOR 1.93; 95% CI 1.09–3.46) were predictive of COVID-19, while cough, running nose and lower neutrophil count were indicative of influenza.

**Figure 2:**
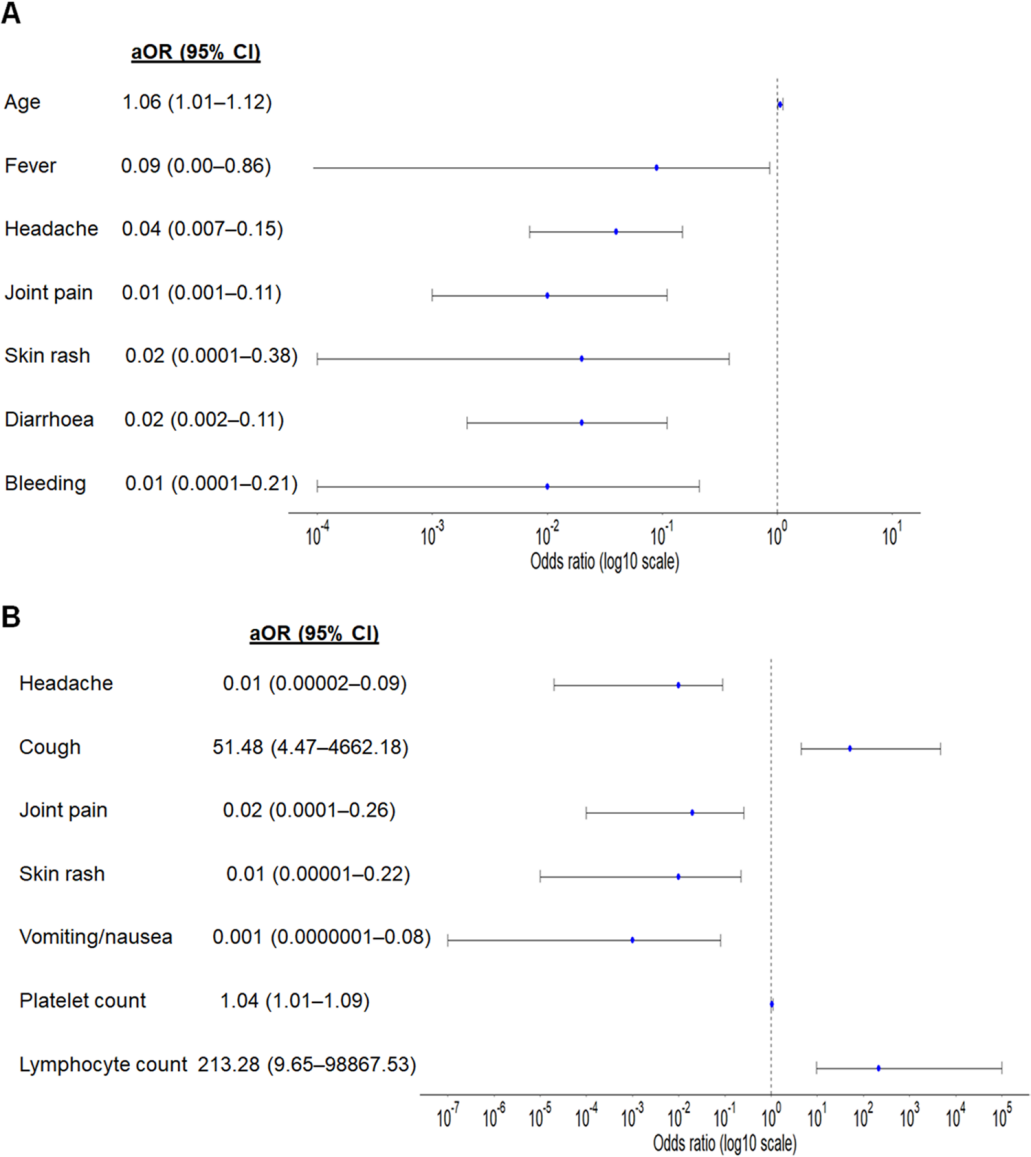
Final covariates in multivariable logistic regression for differentiating COVID-19 versus dengue: (**A**) model 1 on demographics and symptoms, (**B**) model 2 on demographics, symptoms and laboratory parameters. Adjusted odds ratios (aOR) and 95% confidence interval (CI) for having COVID-19 are shown on log10 scale.

Figure 2 shows the multivariable logistic regression analysis differentiating COVID-19 versus dengue. In dengue model 1 containing demographics and symptoms, older age (aOR 1.06; 95% CI 1.01–1.12) increased the odds that the patient had COVID-19, while fever, headache, joint pain, skin rash, vomiting/nauseas and bleeding were indicative of dengue. In dengue model 2 containing demographics, symptoms and laboratory parameters, patients who had cough (aOR 51.48; 95% CI 4.47–4,662.18), higher platelet count (aOR 1.04; 95% CI 1.01–1.09) and higher lymphocyte count (aOR 213.28; 95% CI 9.65–98,867.53) were at increased odds of COVID-19, while cough, headache, joint pain, skin rash and vomiting/nauseas were indicative of dengue.

**Figure 3:**
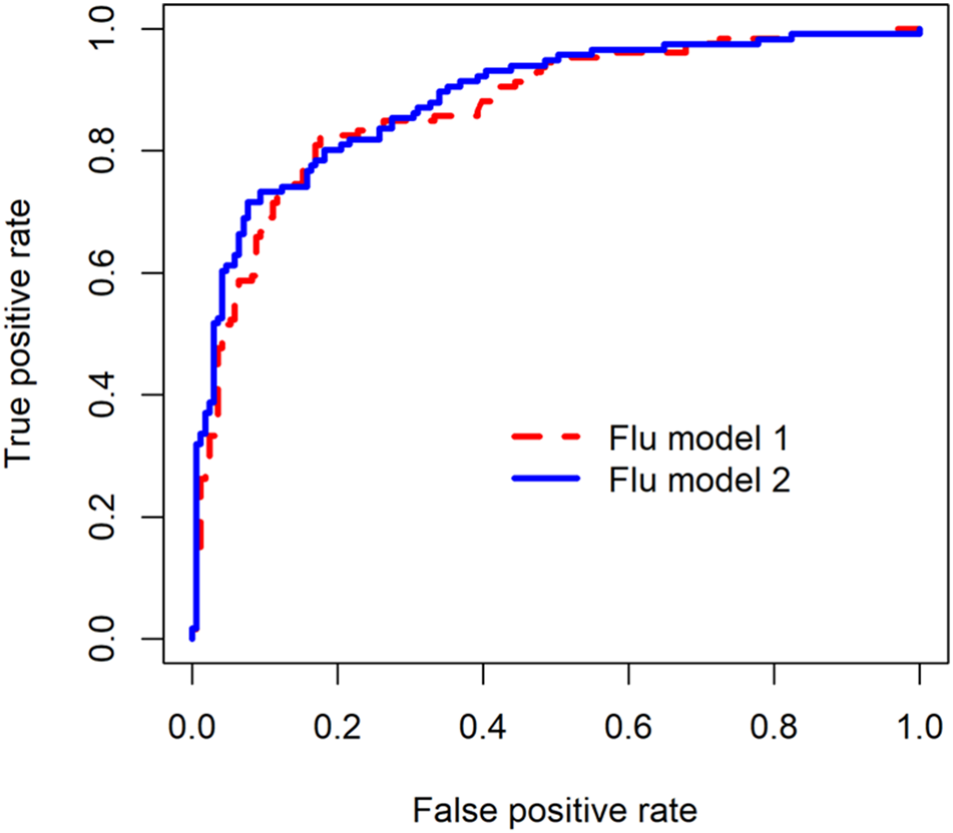
Receiver operating characteristic curves with cross-validation for flu model 1 (demographics and symptoms) and model 2 (demographics, symptoms and laboratory parameters) for differentiating COVID-19 versus influenza.

**Figure 4:**
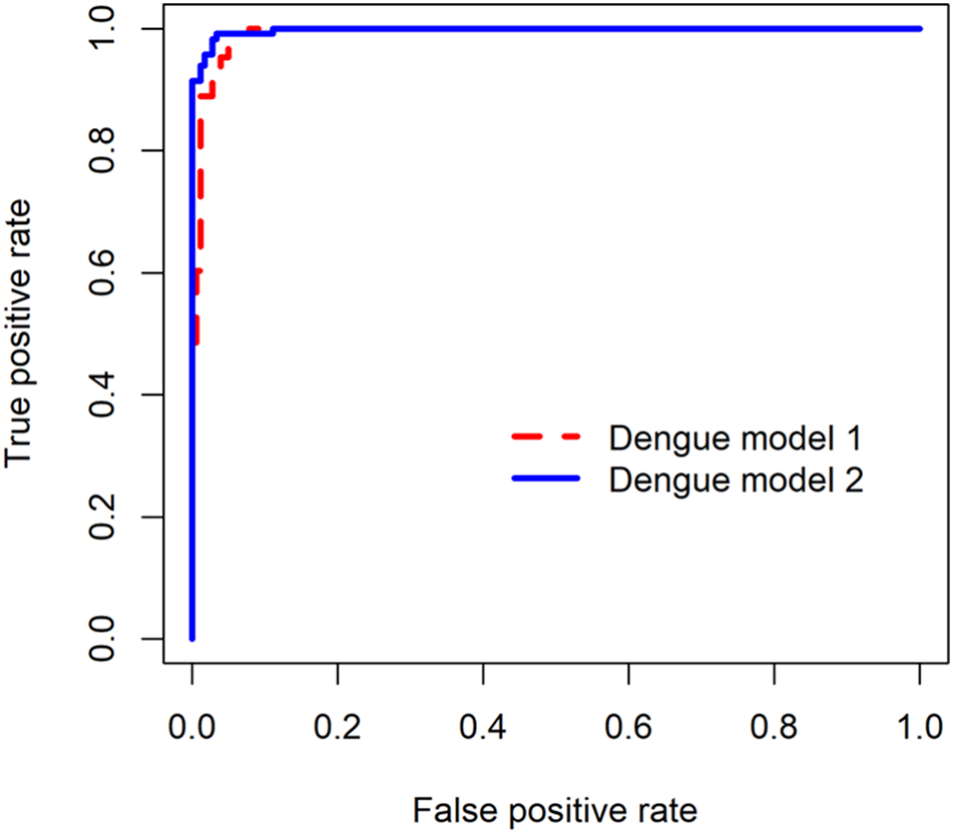
Receiver operating characteristic curves with cross-validation for dengue model 1 (demographics and symptoms) and model 2 (demographics, symptoms and laboratory parameters) for differentiating COVID-19 versus dengue.

The cross-validated AUC of flu model 1 containing demographics and symptoms was 0.871 (95% CI 0.829–0.914), and the cross-validated AUC of flu model 2 which included laboratory parameters in differentiating COVID-19 versus influenza was 0.890 (95% CI 0.851–0.929) (Fig. 3). The cross-validated AUC of dengue model 1 without laboratory parameters and of dengue model 2 which included laboratory parameters for differentiating COVID-19 versus dengue were 0.995 (95% CI 0.989–1.000) and 0.997 (95% CI 0.993–1.000) respectively (Fig. 4).

## Discussion

Early identification of COVID-19 suspected cases is critical for effective surveillance and successful containment of disease spread. Cases who are not diagnosed correctly and in a timely manner may lead to further transmission of the virus^21^. It is imperative to make available a rapid, sensitive, and affordable point-of-care screening test in the primary care setting for early detection of COVID-19^22^. There is possible cross-reactivity between dengue virus and SARS-CoV-2 antibodies, which can lead to false-positive dengue serology among COVID-19 patients and vice versa because of possible similarities between SARS-CoV-2 epitopes in the HR2-domain of the spike-protein and the dengue envelope-protein^23^. In September 2020, the WHO published an interim guidance on the use of SARS-CoV-2 antigen-detecting rapid diagnostic tests (Ag-RDTs) in countries or areas that are experiencing widespread community transmission, where the health system may be over-burdened, and where it may not be possible to test all or any suspect cases by RT-PCR tests. It is recommended to use the Ag-RDT for the suspected symptomatic cases unless the person is a contact of a confirmed COVID-19 case^24^. To ensure that suspected cases are detected as early as possible, primary care physicians who serve as the first point of contact in the healthcare system need a reliable predictive tool to differentiate COVID-19 from other viral infections such as influenza and dengue at first presentation.

In this study, we built logistic regression models using clinical features and simple laboratory investigations to differentiate COVID-19 from influenza and dengue (Figs. 1 and 2). In view of the lower sensitivity of flu model 1 when only symptoms were considered for differentiation of COVID-19 from influenza (Fig. 3), it is important to consider full blood count for further assessment where appropriate. Our study revealed that shortness of breath was the most important symptom for differentiating COVID-19 from influenza. Patients with cough and higher platelet count were more likely to have COVID-19, while headache, joint pain, skin rash and vomiting/nausea were indicative of dengue. These clinical features and simple laboratory parameters can serve as a guide for preliminary screening especially for primary care physicians in Singapore and other tropical countries where dengue is endemic and influenza virus circulates all-year round.

Decision algorithms for differentiating dengue, influenza and other febrile illnesses have been reported before^25^. There were a few studies on prediction models for diagnosis of COVID-19, however, these included measurement of cytokines, computed tomography scan or genome sequencing^26^. Sun et al. had come up with algorithms for estimating the risk of COVID-19 among patients who presented to NCID for SARS-CoV-2 testing, which included a subset of the same cohort of COVID-19 cases in our study, but the duration of symptom onset was not taken into consideration and controls included all SARS-CoV-2 negative patients regardless of final diagnosis^27^. In the study by Sun et al., the AUC of the logistic regression model that predicted COVID-19 was 0.65 when only demographics and clinical variables were considered, whereas the models with laboratory and exposure-risk variables included had higher AUCs of 0.88 to 0.91^27^.

As our study relied on simple parameters to differentiate COVID-19 from influenza and dengue, the predictors identified from the multivariable logistic regression models may guide primary care physicians, who serve as the first point of contact with the healthcare system, in deciding who should be tested with RT-PCR tests or Ag-RDTs especially in the absence of travel history to high-risk countries and epidemiological links^16^. In countries and remote regions where confirmatory diagnostic capabilities are limited^28^, clinical features and simple laboratory parameters identified in this study may be used as one of the criteria for isolation of suspected cases to prevent further transmission arising from prolonged delay from symptom onset to isolation^29^.

There are a few limitations in our study. We included patients who presented to hospital within 5 days of symptom onset, hence the findings may not be applicable to those presented late for medical care. Patients who were not suspected or sufficiently ill to be referred to hospitals were excluded. The patients in our study were recruited in three different periods as it was not feasible to recruit influenza and dengue patients during the COVID-19 outbreak in 2020; (i) there was a drastic decline in influenza activity and (ii) non-COVID-19 related research activities were put on hold to minimize the risk of COVID-19 transmission. There may be differences in clinical features and laboratory parameters at presentation due to the predominant strains of SARS-CoV-2 virus among COVID-19 patients, virus serotypes among dengue patients, and virus strains among influenza patients circulating at different time points. Few COVID-19 cases co-infected with dengue had been reported thus far in Singapore, Thailand and French island of Mayotte^30–32^. Case series of pneumonia patients co-infected with COVID-19 and influenza were also reported in China^33^. In Singapore, after the implementation of public health measures for COVID-19, a marked decline in influenza activity had been reported^34^. There is an urgent need for clinical, epidemiological and laboratory studies to be undertaken to better understand the interactions between COVID-19, influenza and dengue.

In conclusion, we have shown that multivariable models based on clinical features and simple laboratory markers for differentiating COVID-19 from influenza and dengue, which possess good predictive performance can serve as a useful tool for primary care physicians to determine if further investigations or referrals would be required. The findings from our study would need to be further validated, so as to address the knowledge gaps of the ongoing COVID-19 pandemic.

## Supplementary Information

Supplementary Information.

## Data Availability

The datasets analysed during the current study are available from the corresponding author on reasonable request.
