# Supplementary material for: Differentiating coronavirus disease 2019 (COVID-19) from influenza and dengue
Source: Sci Rep. 2021 Oct 5;11:19713. doi: 10.1038/s41598-021-99027-z (PMC8492678; doi:10.1038/s41598-021-99027-z)
Supplement: Supplementary file 1 — Supplementary Information. [file 41598_2021_99027_MOESM1_ESM.docx]

**Supplementary Table File**

**Differentiating coronavirus disease 2019 (COVID-19) from influenza and dengue**

Tun-Linn Thein^1^^, Li Wei Ang^1^^, Barnaby Edward Young^1,2,3^, Mark IC Chen^1,4^, Yee-Sin Leo^1,2,3,4,5*^, David Chien Lye^1,2,3,5*^

^1^ National Centre for Infectious Diseases, 16 Jalan Tan Tock Seng, 308442, Singapore
^2^ Department of Infectious Diseases, Tan Tock Seng Hospital, 11 Jalan Tan Tock Seng,
308433, Singapore
^3^ Lee Kong Chian School of Medicine, Nanyang Technological University, 11 Mandalay
Road, 308232, Singapore
^4^ Saw Swee Hock School of Public Health, National University of Singapore and National
University Health System, 12 Science Drive 2, #10-01, 117549, Singapore

^5^ Yong Loo Lin School of Medicine, National University of Singapore and National University Health System, 10 Medical Drive, 117597, Singapore

^These authors contributed equally to the manuscript

**^*^Corresponding author:** Prof. Yee-Sin Leo

National Centre for Infectious Diseases

16 Jalan Tan Tock Seng

Singapore 308442

Telephone: +65 63577911, Fax: +65 65115083

Email: [Yee_Sin_Leo@ncid.sg](mailto:Yee_Sin_Leo@ncid.sg)

**Supplementary Table S1. Visual comparison of percentage of COVID-19, influenza and dengue patients by symptoms at presentation**

| **Symptoms** | **COVID-19** | **Influenza** | **Dengue** |
| --- | --- | --- | --- |
| Fever | ++++ | +++++ | +++++ |
| Cough | ++++ | +++++ | ++ |
| Sore throat | ++ | +++ | ++ |
| Running nose | ++ | +++ | + |
| Sputum | ++ |  |  |
| Shortness of breath | + | + | + |
| Headache | + | + | +++++ |
| Abdominal pain | + | + | + |
| Chest pain | + |  | + |
| Joint pain | + |  | ++++ |
| Muscle ache | + | + | ++++ |
| Vomiting/nausea | + | + | ++++ |
| Diarrhoea | + | + | ++ |
| Fatigue/malaise | + | + | +++++ |
| Skin rash | 0 |  | ++ |
| Bleeding | 0 |  | ++ |
| Conjunctivitis | 0 |  | + |

‘+’ sign refers to more than zero to 20% having the symptom, ‘+++++’ sign refers to more than 80% to 100% having the symptom, ‘0’ indicates no one has the specific symptom.

Blank means the specific symptom data is not available.

COVID-19, coronavirus disease 2019.
